# Bioprotective and Technological Roles of Lactic Acid Bacteria in Reduced-Sodium Fermented Sausages

**DOI:** 10.3390/foods14213758

**Published:** 2025-11-01

**Authors:** Marcello Lima Bertuci, Camila Vespúcio Bis Souza, Carlos Alberto Alves, Svetoslav Dimitrov Todorov, Ana Lúcia Barretto Penna, Andrea Carla da Silva Barretto

**Affiliations:** 1 Department of Food Technology and Engineering, UNESP—São Paulo State University, 2265 Cristóvão Colombo Street, São José do Rio Preto 15054-000, SP, Brazil; camila.bis@unesp.br (C.V.B.S.); carlos.junior@unesp.br (C.A.A.J.); ana.lb.penna@unesp.br (A.L.B.P.); andrea.carla@unesp.br (A.C.d.S.B.); 2 ProBacLab, Laboratório de Microbiologia de Alimentos, Departamento de Alimentos e Nutrição Experimental, Food Research Center, Faculdade de Ciências Farmacêuticas, Universidade de São Paulo, São Paulo 05508-000, SP, Brazil; slavi310570@abv.bg

**Keywords:** sodium reduction, product development, healthier products, food safety, biopreservation, bacteriocins

## Abstract

Fermented sausages are popular worldwide due to their sensory and nutritional characteristics, as well as their convenience for storage and consumption. The production and consumption of meat products are associated with negative impacts from the risks of high sodium intake, such as cardiovascular disease and hypertension. Salt (NaCl) plays an important role in the preservation, water loss during drying, reduction in water activity, and sensory characteristics of meat and other fermented food products. NaCl reduction is considered a challenge because it affects the sensory properties of meat and can compromise the safety and microbiological parameters related to the spoilage of the fermented meat product. The use of microorganisms, such as LAB, has been studied as an innovative way to substitute traditional preservatives. They produce various metabolites, including bioactive and antimicrobial substances that are actively involved in health benefits and guarantee the safety of meat products. These natural substances produced by bacteria extend shelf life by inhibiting spoilage and pathogenic microorganisms. This review discusses the potential application of lactic acid bacteria in the reformulation of fermented sausages, challenges, and beneficial effects on sensorial, safety, and health properties.

## 1. Introduction

The food industry is an iconic example of the relationship between the development of fundamental science and the needs of society. In the post-war periods, the objective of the food industry was to provide sufficient food to the population, and this was related to the development of different preservation techniques. However, with time, due to the increasing consumer demand for healthier products with fewer additives (clean-label products), researchers and development team members of the food (including meat production and processing) industries have been challenged to explore some alternatives to innovate these products through product reformulations. One of the principal tasks was to reduce the use of NaCl as one of the principal preservatives for numerous food products after being used for millennia. The reduction in NaCl levels can be considered one of the principal challenges in reforming meat products. This can influence the water activity (a_w_) and be directly and indirectly related not only to potential spoilage and pathogen growth but also to the loss of technological and sensory qualities of the final product [1].

Salt is a crucial ingredient in sausage fermentation, significantly influencing sensory characteristics such as color, flavor, and texture. It influences moisture, which affects the juiciness and tenderness of the final product. In addition, different types of salts (including NaCl) act as a preservative, contributing to the extension of the shelf life of food products [2]. In contrast, moderate NaCl consumption (2 g daily) is essential for the normal functioning of the human body and contributes to maintaining good health [3,4].

The reduction of a_w_ through the addition of salts and solutes helps reduce or inhibit the activity of microorganisms and enzymes, thereby improving product preservation. Meat products with reduced NaCl content exhibit a shorter shelf life because NaCl plays a critical role in preservation by inhibiting spoilage and pathogenic microorganisms through its bacteriostatic effect, primarily by lowering a_w_ [5]. In addition, it can be combined with other preservation methods, such as temperature control, acidification, and fermentation. Various antimicrobial compounds produced by LAB during fermentation have been studied with promising results for potential future applications in meat products [6].

The use of strains capable of producing beneficial metabolites, exhibiting compatible growth characteristics, and having distinct advantages in metabolic performance can significantly enhance the safety and quality of low-sodium fermented foods. The beneficial effects of different strains in low-sodium fermented foods extend beyond simple additive interactions. The complex interactions between strains and their environment can play a crucial role in determining the final product’s overall quality [7].

Considering the increasing concern about excessive NaCl intake in the diet, the food industry and researchers in the field are exploring strategies to reduce the NaCl content in traditional meat products. This can be achieved by replacing some amount of salt with lower NaCl alternatives or by employing processing technologies that diminish the reliance on salt for preservation [8]. However, any NaCl reduction must be evaluated in advance to ensure that the product meets food safety requirements and maintains sensory characteristics [9].

Biopreservation by LAB fermentation has been increasing, especially in animal-origin products, with a significant presence in dairy products (yogurt, cheese, fermented milk) and in fermented sausages in recent years [10,11]. The biopreservation method is based on the utilization of natural substances derived from bacteria. Fermentation is one of the most common methods, in which beneficial microorganisms are cultivated on food under controlled conditions to inhibit the growth of spoilage-causing microorganisms [12,13].

LAB are beneficial microorganisms that contribute to improving the quality and safety of meat products [14]. They facilitate rapid acidification, resulting in lower pH values that define the fermentation endpoint. This enhances microbial stability by inhibiting the activity of spoilage organisms and pathogenic bacteria. Moreover, the acidic environment accelerates nitrite reduction, promoting faster color development and stabilization. LAB produces a range of compounds that contribute to flavor development and exhibit antimicrobial activity. These processes trigger physical, chemical, and biochemical changes that shape the products’ distinctive sensory attributes throughout fermentation and ripening [15,16].

Although several factors (such as low pH and a_w_, salt and nitrites, chemical preservatives, and starter cultures) collaborate to ensure that the meat product does not pose a risk to safe consumption, certain pathogenic microorganisms, such as *Listeria monocytogenes*, can adapt to the environment of fermented sausages, compromising the product’s safety. Therefore, additional barriers, such as the use of biopreservatives, different packaging technologies, and a reduction in a_w_, may be necessary [17].

In biopreservation processes, LAB contributes to inhibiting the growth of *Listeria* spp. (especially *L. monocytogenes*) and other spoilage and food-borne pathogens by producing lactic acid, synthesizing inhibitory compounds, competing for nutrients, and reducing a_w_ [18]. The inhibitory influence of the representatives from the former *Lactobacillus* genus, reclassified into 23 new genera in 2020 [19,20], on the growth of *L. monocytogenes* and food safety is well documented. However, understanding that using biopreservative cultures is not a panacea for controlling spoilage and food-borne pathogens is crucial. Instead, it is an integral part of a broader set of microbial growth management strategies [21].

In the last few decades, the improvement of quality of life, development of all branches of the fundamental and applied sciences, revolution in communication and information sharing, and consumer demand for healthier products have become key factors in the development of new strategies in the formulation of food products, including the rediscovery of the beneficial roles of LAB in improving food safety. in fermented sausages is the reduction of sodium chloride and/or nitrite, driven by consumer demand for healthier options, without compromising sensory quality or microbial safety. Concurrently, the application of probiotics has highlighted the potential of LAB to confer health benefits to consumers. Despite these advances, there is limited understanding of how specific LAB strains can simultaneously improve safety, sensory attributes, and functional properties in reduced-sodium fermented sausages. Therefore, this review aims to critically examine the technological, antimicrobial, and potential probiotic functions of LAB in sodium-reduced fermented sausages, highlighting challenges and future research directions [22].

## 2. Innovative Reformulation of Fermented Meat Sausages

### 2.1. Sodium Chloride Reduction Strategies

**Figure 1:**
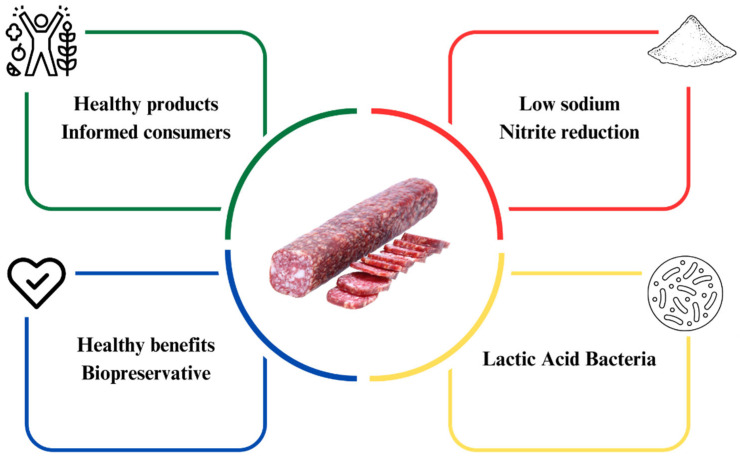
Reformulation of fermented sausages.

Fermented sausages constitute a diverse category of meat-based foods that undergo controlled microbial fermentation to enhance preservation, safety, and sensory attributes. This category encompasses various items, including dry-cured hams, fermented sausages, and other regional or traditional products. Fermented meat sausages, a specific subset within this category, are characterized by the comminution and mixing of meat and fat, combined with salt, curing agents, spices, and starter cultures, followed by regulated fermentation, drying, and ripening processes. These processes induce complex physicochemical and microbiological changes that result in distinct texture, flavor development, and microbial stability. Distinguishing fermented meat sausages from the broader class of fermented sausages is important, as the former represents a specific product type with unique processing parameters and quality attributes. In recent years, the food industry has endeavored to enhance the appeal of these meat products to consumers, making them healthier without compromising their sensory characteristics [23]. However, this reformulation poses a challenge as it seeks to preserve technological and sensory characteristics, including texture, flavor, color, aroma, and microbiological quality. These aspects directly influence the shelf life of the product [24]. The reformulation of fermented meat sausages is summarized in Figure 1, which schematically illustrates the main technological and compositional adjustments involved in developing healthier products, including sodium and nitrite reduction strategies and their effects on product quality and safety.

The addition of NaCl to fermented sausage formulation acts as a preservative agent by reducing the a_w_ in meat, creating a less favorable environment for spoilage microorganism growth. It also enhances the flavor and preserves the meat texture [25]. Salt acts on the osmotic balance, promoting water exchange between meat cells and the surrounding environment. This influences water loss from the meat cells to the outside, promoting controlled dehydration during drying [26]. Fresh meats and uncured sausages have high a_w_, very close to 1.0, which supports microbial growth. In contrast, products such as ham, bacon, and cured sausages (salami) exhibit moderate a_w_ (0.85–0.95), reduced by the addition of salt, nitrites, and nitrates, which helps to inhibit microbial development. Products such as salami, pepperoni, and beef jerky have low a_w_ (typically below 0.85) due to moisture loss during drying. This significant reduction in a_w_ directly impacts meat product quality, safety, stability, and shelf life. Most pathogenic bacteria, including *Salmonella* spp., *Listeria* spp., and *Escherichia coli*, require a_w_ levels of >0.90 for growth and are inhibited at lower aw levels. Yeasts can proliferate at lower a_w_ levels (0.85), whereas fungi and molds can thrive in even drier conditions (0.70) [27].

Reformulation by reducing NaCl meets the demand for healthier products. Approximately 20–30% of an individual’s dietary sodium intake is derived from meat products. Sodium, when consumed in large quantities exceeding 5 g per day according to the World Health Organization (WHO), can cause health risks such as cardiovascular problems and hypertension [28,29]. NaCl facilitates the extraction and solubilization of myofibrillar proteins, which are essential for water retention within the matrix of meat products, directly affecting their texture and yield. Thus, a reduction in NaCl concentration leads to a decrease in technological and sensorial properties and can compromise food safety [30].

There are various strategies for reducing the NaCl content of fermented sausages, including direct reduction. Substituting NaCl with metallic salts, such as potassium chloride, calcium chloride, and magnesium chloride, along with the incorporation of flavor enhancers, such as monosodium glutamate and nucleotides, can aid in lowering the NaCl content in meat products while preserving their sensory quality and safety. The use of potassium chloride is the most employed. However, the heightened use of metallic salts may lead to undesirable side effects, including a bitter or metallic taste and texture degradation [31].

Many studies on NaCl reduction in meat products are available in the literature. Corral [32] and Chen [33] have reported a 30–50% reduction in NaCl in fermented sausages while preserving their sensory characteristics. However, the interaction between the product matrix and the peptides, as well as processing conditions such as temperature and pH, can significantly impact the ability of the peptides to modulate the salty taste. The mechanism underlying this perception involves interactions with taste receptors, modulation of salt sensation, effects on salt release, and flavor potentiation. Peptides can influence the overall flavor profile of the product by balancing saltiness with other taste sensations, such as umami. This interplay can alter the perceived salty taste intensity [34].

### 2.2. Nitrate and Nitrite Reduction Strategies

The addition of sodium nitrite and sodium nitrate to fermented meat sausages is responsible for many functions, including color enhancement, providing an antimicrobial effect by inhibiting the growth of pathogenic and spoilage bacteria, imparting antioxidant properties, and contributing to the formation of cured product characteristics [35]. However, despite the numerous positive aspects of their application, the use of nitrite and nitrate has raised several consumer health concerns due to the formation of carcinogenic N-nitrous compounds, specifically nitrosamines. These compounds can be produced in both food matrices and the human body [36]. One of the reasons for attributing these risks is heat treatment, which enhances these compounds’ formation. However, this concern does not apply to fermented meat sausages, as they do not require heat treatment [37].

The use of these preservatives is highly regulated in the food industry, with well-defined limits for their incorporation in product preparation. These limitations aim to prevent the consumption of excessive amounts of nitrite and nitrate [38]. Additionally, natural food preservation methods, such as fermentation, are considered favorable because they do not negatively affect consumers’ health and may have a smaller impact on nutritional and sensory properties.

For instance, Chen [39] used *Lactiplantibacillus plantarum* P2 isolated from fermented sauerkraut and observed its potential to efficiently eradicate peroxide free radicals and degrade sodium nitrite. Reducing the nitrite content in the product using LAB is emphasized as an important production strategy. Moreover, selenium supplementation displayed antioxidant capacity, showing its potential application in fermentation process and new opportunities for producing functional foods.

Zhu [40] investigated the partial replacement of nitrite in Chinese fermented sausage by *Lpb. plantarum*. LAB enhanced the gel-forming properties and increased viscoelasticity. Additionally, *Lpb. plantarum* application enhanced the reduction in the risk of biogenic amines, attributed to a decrease in tyramine levels. LAB played a role in improving the color and gel properties and reducing the levels of nitrate and biogenic amines.

## 3. Role of Lactic Acid Bacteria in Fermented Sausage

Lactic acid bacteria are present in diverse niches, including food of both animal and plant origin, human microbiota, and the environment. LAB are described as Gram-positive microorganisms that have a rod or coccobacilli shape, are devoid of cytochromes, are non-spore-forming microorganisms, and are catalase negative, with noted exceptions (pseudo catalase activity). The oxygen requirements are very diverse, and the bacteria can be classified as anaerobic or facultative anaerobic [41]. Carbohydrate metabolism can be either homofermentative, resulting in the production of organic acids, mainly lactic acid, or heterofermentative, resulting in the production of organic acids, such as lactic acid, carbon dioxide, and other fermentation products, such as alcohol and carbonyl compounds, such as diacetyl, acetaldehyde, acetoin, and 2-butanone [42].

In fermented meat sausage, glucose or other sugars present in muscle tissues are utilized as substrates for bacterial energy production. Moreover, depending on the metabolite properties of the applied species/strains, a variety of organic acids (such as lactic, acetic, and propionic acids) can be produced, which contribute to the acidification of the environment. LAB also exerts a range of adverse effects on other microorganisms, including competition for nutrients and production of inhibitory compounds. Additionally, LAB are known to produce an extended spectrum of other substances, including organic metabolites such as polymers, sweeteners, nutraceuticals, aromatic compounds, various enzymes, and antimicrobial compounds [43,44,45]. Fermentation processes not only influence the physicochemical parameters of meat products but also serve as the foundation for the functional activities of LAB.

### 3.1. Biopreservative Compounds Produced by LAB

Beyond their fundamental role in acidification and fermentation, LAB also synthesize a variety of biopreservative compounds such as bacteriocins, organic acids, and hydrogen peroxide that contribute significantly to microbial control and food safety. Biopreservative compounds, including organic acids, hydrogen peroxide, antifungal compounds, bacteriocins, and bacteriocin-like inhibitory substances (BLIS), inhibit the growth of food-borne pathogenic and spoilage microorganisms, thereby enhancing product safety [40,46]. For instance, biopreservative compounds can also play a role in a distinct approach to barrier technology, where they are strategically combined with other barriers to address food spoilage without compromising starter or adjunct cultures. This approach ensures that the desired fermentation process proceeds, maintaining both the quality and safety of the food product. The rationale behind adopting this integrated approach is the potential to subject unwanted microorganisms to multiple obstacles that inhibit their growth and survival. Moreover, if there is synergy, using smaller quantities of preserving agents and/or reduced levels of technological treatment becomes feasible [47]. The application LAB that produces biopreservatives meets consumer demand for healthier products with a reduction in chemical products.

Currently, the importance of the quality and safety of fermented sausages is becoming increasingly evident, being a topic of prominent studies. Additionally, several challenges must be addressed for the effective application of bacteriocins, including their stability in different formulations, the influence of environmental factors such as pH and substrate availability, and the need for rigorous validation in vivo and in vitro testing [48,49].

**Figure 2:**
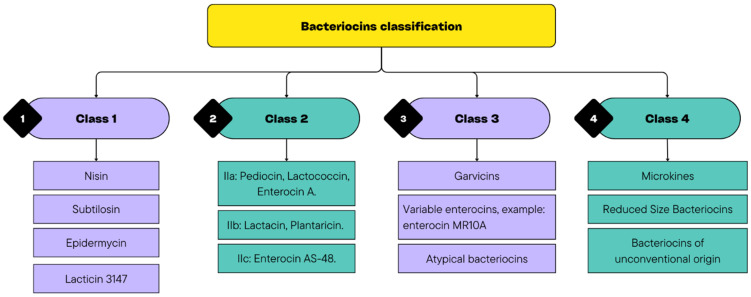
Diversity of Bacteriocin Classes.

Bacteriocins can help overcome these obstacles through their broad-spectrum antimicrobial activity, resistance to proteolytic degradation under certain conditions, and synergistic interactions with other preservation strategies. They consist of antimicrobial peptides that can inhibit spoilage and pathogenic bacterial species. Moreover, in recent decades, it was suggested that bacteriocins may have activity even against some yeasts and viruses [50]. They are classified into four main classes, with Classes 1 and 2 being the most relevant in food applications (Figure 2). Class 1 includes antibiotics, whereas Class 2 consists of low-molecular-weight non-lantibiotic bacteriocins, which are further subdivided into subgroups (IIa, IIb, and IIc). In addition, Class 3 comprises unmodified low-molecular-weight peptides, such as microcins, and Class 4 encompasses bacteriocins produced by *E. coli*, which have distinct characteristics that do not align with those of the previous classes, such as colicin [51,52].

Their mechanisms include cell wall permeabilization, vital protein inhibition, pore formation, interference with DNA replication, and immune system modulation. It is noteworthy that the bacteria that produce these bacteriocins possess specific immune mechanisms that protect them from their effects. These substances are widely recognized as safe, exhibiting no cytotoxic activity on eukaryotic cells (such as nisin in most of the studied cases), being inactivated by digestive enzymes such as proteases, and having minimal impact on the intestinal microbiota [42,53]. The ideal characteristic of a biopreservative agent is to exhibit specific antimicrobial activity solely against the target pathogenic or spoilage microorganism without adversely affecting the product and the microbiota of consumers [54].

BLIS refers to a heterogeneous group of antimicrobial peptides produced by bacteria, primarily LAB, that exhibit inhibitory activity against spoilage microorganisms and foodborne pathogens [55]. Unlike well-characterized bacteriocins, BLIS have not been fully defined in terms of their amino acid composition and biochemical properties, which distinguishes them in both definition and applicability. Despite these limitations, BLIS shows potential as a natural biopreservative, displaying bactericidal or bacteriostatic effects against Gram-positive and, in some cases, Gram-negative bacteria [56]. Interactions with food matrix components such as lipids and proteins, as well as susceptibility to proteolytic degradation, may hinder their antimicrobial effectiveness [57,58]. However, combinatory strategies such as the co-application of BLIS with bacteriocins or other antimicrobial metabolites have proven effective in enhancing their activity, particularly in high-fat meat products where isolated bacteriocins often exhibit reduced efficacy. Therefore, BLIS represents a promising alternative within the concept of postbiotics, contributing to food microbiological safety through a natural and technologically viable approach [59].

As examples, pediocin from *P. acidilactici*, nisin from *L. lactis*, and carnobacteriocin BM1, carnocyclin A, and piscicolin 126 from *C. maltaromaticum* are some of the few commercial bacteriocins approved by the American Food and Drug Administration (FDA). These bacteriocins are classified as Generally Recognized as Safe for use as food preservatives or additives. Moreover, their antimicrobial activity and specificity against pathogens can be enhanced through bioengineering. Because of their relatively simple biosynthetic pathways, their genetic determinants can be manipulated with minimal challenges [60]. These combined mechanisms effectively hinder the growth of undesirable microorganisms in fermented foods, thereby contributing to their preservation and safety [61]. Various strains of LAB, including *L. sakei*, *Lpb. plantarum*, *L. animalis*, and *L. curvatus*, can function as effective bioprotective agents in meat and meat products [62].

Bacteriocins are classified into three major classes based on their structural, biochemical, and genetic characteristics: class I (lantibiotics), class II (unmodified heat-stable peptides), and class III (large heat-labile peptides). Class I bacteriocins, such as nisin, are peptides smaller than 5 kDa that contain unusual amino acids, such as lanthionine and methyl-lanthionine, which confer high thermal stability and resistance to proteolytic enzymes. Class II bacteriocins, exemplified by pediocin PA-1 (class IIa), are small (<10 kDa), cationic, hydrophobic, and heat-stable peptides that form pores in the target cell membrane and exhibit strong antilisterial activity. In contrast, class III bacteriocins, such as helveticin J, are larger molecules (>30 kDa) and are generally thermolabile, exerting their antibacterial effects primarily through enzymatic degradation of the bacterial cell wall. In meat products, class I and II bacteriocins have demonstrated greater efficacy against Gram-positive pathogens, such as *Lactobacillus monocytogenes* and *Clostridium botulinum*, particularly when used in combination with other hurdle technologies. Nisin is widely used in processed meat products because of its broad-spectrum antimicrobial activity and stability under adverse conditions. Meanwhile, pediocin PA-1 is especially effective in fermented sausages because of its high specificity against *Listeria* species [63,64].

Lactocin MM4, a novel bacteriocin produced by *Companilactobacillus alimentarius* FM-MM4, showed a molecular mass and N-terminal sequence of 1104.58 Da (QGVGPLGQGHR) and low homology with known class II bacteriocins. This bacteriocin exhibited broad-spectrum antimicrobial activity against Gram-positive and -negative pathogens, as well as several yeasts. It demonstrated high thermal stability, retaining 84.7% of its antimicrobial activity after exposure to 121 °C for 15 min, and remained effective at acidic pH (2–5). Hu [65] reported that proteolytic enzymes completely inactivated lactocin MM4, whereas lipase and amylase had no effect, reinforcing its potential application in food preservation.

Due to the non-sterility of the raw materials and ingredients used in the production of fermented sausages, there exists the potential for contamination by food-borne pathogens. This allows these microorganisms to proliferate during the fermentation process. An alarming example of a foodborne pathogen is *L. monocytogenes*, which poses a potentially fatal threat to public health [63]. While acidification plays a central role in stabilizing fermented sausages, the bioprotective capacity of LAB extends far beyond pH reduction, involving the production of metabolites with antimicrobial activity against spoilage and pathogenic microorganisms.

### 3.2. LAB for the Biopreservation of Fermented Sausages

**Figure 3:**
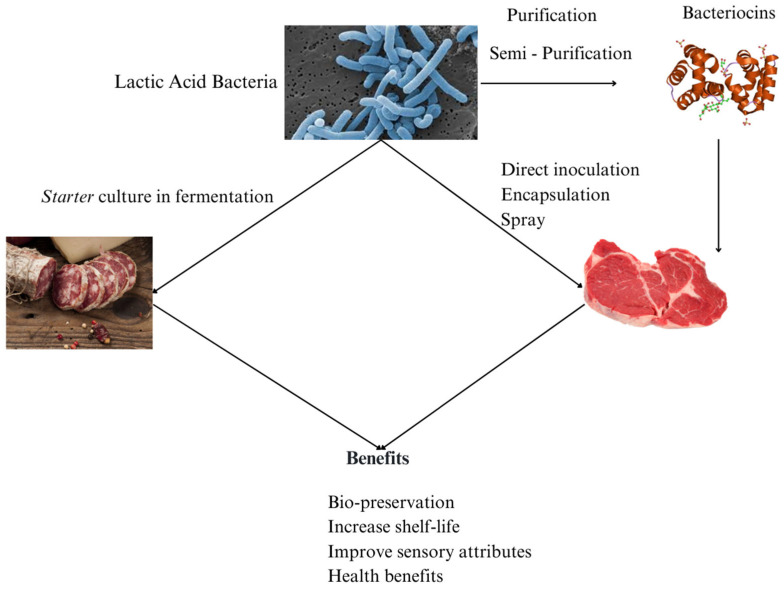
Lactic acid bacteria application in meat products.

In addition to lowering pH and promoting fermentation stability, LAB produce a wide range of antimicrobial substances that enhance food safety and extend shelf life. Certain compounds are particularly noteworthy to the food industry because of their capacity to minimize lipid oxidation through a combination of effects, including acidification of the environment, oxygen consumption, production of antioxidant substances, and modulation of enzymes. These mechanisms collectively contribute to product quality preservation and shelf life extension [64]. Figure 3 provides a schematic overview of the different approaches for applying lactic acid bacteria in meat products, highlighting their multifunctional roles as starter, protective, and probiotic cultures contributing to safety, shelf life, and sensory quality.

The application of different LAB species in fermented meat sausages—*Lpb. plantarum*, *L. casei*, *L. paracasei, Ltb. sakei*, and *L. rhamnosus*—has been evaluated as a protective culture. These LAB strains produce a wide range of antimicrobial compounds, including organic acids (e.g., lactic acid and acetic acid), bacteriocins, hydrogen peroxide, and enzymes, which contribute to their bioprotective effects. This biopreservation strategy harnesses the natural metabolic activities of LAB to suppress the growth of spoilage microorganisms and pathogenic bacteria, such as *Listeria monocytogenes*, *Escherichia coli*, and *Salmonella*, thereby extending the shelf life and safety of meat products [65,66].

Several studies [67,68,69] have demonstrated the capability of *L. monocytogenes* to grow and proliferate in “stress” conditions, such as low temperatures, high salinity levels, and low pH, and the ability to form biofilms, among other challenging situations that typically inhibit microbiological growth. Consequently, *L. monocytogenes* may be present in food processing environments, posing a potential contamination risk. Numerous studies have associated LAB in meat products with enhanced fermentation, product characteristics, and preservation (Table 1).

The positive results indicate that the integration of microorganisms into fermented meat sausages enhances their ability to extend the shelf life of foods. The applications of microorganisms influence technological characteristics such as pH, water activity, and inhibition of pathogenic and spoilage microorganisms [70]. Several studies have focused on the application of LAB to fermented sausages. The variability in the technological and bioprotective performance of LAB strains can be attributed to their strain specific metabolic profiles, including differences in acidification kinetics, proteolytic and lipolytic systems, and bacteriocin production capacity are demonstrated in Table 1, while bacteriocin production capacity is presented in Table 2.

**Table 1:** Application of lactic acid bacteria in reformulated fermented sausages and their challenges.

Lactic Acid Bacteria	Objective	Results	Reference
Nitrite reduction/free			
*Lactobacillus lactis* MP11, *P. acidilactici* MP14, *L. salivarius* MP02c, and *P. acidilactici* B-LC-20	Anti-listerial activity of selected bacteriocin-producing LAB in vitro and in a fermented sausage model developed with or without small sodium nitrite concentrations	*P. acidilactici* MP14 reduced *Listeria* counts in a nitrite-reduced environment. The reduction observed was similar to that caused by the commercial strain *P. acidilactici* B-LC-20, both in vitro and in the meat model.	[71]
*Weissella cibaria* X31 and *Weissella confusa* L2	The effects of the *Weissella* species as a starter on the physicochemical and proteolytic properties of low-nitrite dry-fermented sausage were evaluated.	*W. cibaria* X31 and *W. confusa* L2 resulted in high redness values in the final product. Residual nitrite levels were reduced by 95–97%. Both strains suppressed the growth of *S. enterica*.	[72]
*Lpb. plantarum*	The positive effect of *Lpb. plantarum* on the reduction of nitrate and biogenic amine content, color, and gel property of fermented sausages	In Chinese fermented sausages, the combination of low levels of sodium nitrite and *Lpb. plantarum* resulted in reduced residual nitrite and biogenic amine levels, and increased color and gel properties.	[40]
*Mammaliicoccus sciuri* IMDO-S72 and *Ltb. sakei* CTC 494	Monitoring the growth of *C. botulinum*-like strains in group I during the production of fermented sausages without added nitrate and nitrite salts	The addition of *M. sciuri* IMDO-S72 as an anticlostridial starter culture did not result in any additional antibacterial effect.	[73]
*Lactobacillus* MPKL03 and MPKL04	The ability of nitrite-reducing performance under different production and processing conditions of two LABs isolated from Sichuan traditional sausage	MPKL03 and MPKL04 reduced residual nitrite levels and influenced microbial counts in traditional Sichuan sausages.	[74]
Low sodium			
*Ltb. curvatus*, *Ltb. sakei*, *Weissella hellenica*, and *Lpb. plantarum*.	The potential taste-compensating role of these LAB strains in reduced-salt dry sausage was evaluated.	Free amino acids (FAAs) and organic acids were detected in reduced-salt dry sausage, influencing measurable taste-related parameters.	[75]
*Ltb. curvatus* SYS29, *Ltb. sakei* HRB10, *W. hellenica* HRB6, and *Lpb. plantarum* MDJ2	investigate the compensative role of four autochthonous LAB strains in the physicochemical properties and taste profiles of dry sausages substituted with NaCl	In dry sausages with 40% NaCl substituted by KCl, inoculation with LAB starter cultures resulted in faster acidification, increased water loss, and higher levels of FAAs and organic acids.	[76]
*Lpb. plantarum* LPL-1	Potential application of the strain as a starter culture for low-salt fermented sausage production	LPL-1 reduced microbial counts associated with spoilage and pathogens in low-salt fermented sausages.	[77]
*Lpb. plantarum* CRL681, *Ltb. curvatus* CRL705, *Ltb. sakei* CRL1862 and *Enterococcus mundtii* CRL35	Biochemical analysis of the production of small peptides and free amino acids by different LAB strains in a sausage model with reduced sodium content	The starter combination *E. mundtii* CRL35 + *S. vitulinus* GV318 showed the highest levels of peptides. *L. sakei* CRL1862 + *S. vitulinus* GV318 resulted in increased amino acid production.	[78]
*Bifidobacterium animalis* ssp. *lactic BB-12*	Produce Italian salami with encapsulated probiotic microparticles and reduced curing salt, and evaluate the product’s physicochemical and sensory characteristics and probiotic viability.	The addition of microparticles containing *Bifidobacterium* BB-12 combined with reduced curing salt did not significantly change physicochemical parameters, lipid oxidation, color parameters (a * and b *), texture profile, fatty acid profile, or organic acid content. In sensory evaluation, treatment B2 (curing salt reduction + encapsulated BB-12) received the highest acceptance scores.	[79]

**Table 2:** Bacteriocin-producing lactic acid bacteria in fermented sausages.

Bacteriocin-Producing Strain	Objective	Results	Reference
*P. acidilactici* (B-L20SafePro^®^, Hansen).	Bioprotection cultures for dry-fermented salami to control *L. monocytogenes* growth during the manufacturing process	The bioprotection culture inhibited the growth of *L. monocytogenes* in dry fermented sausages throughout the ripening/drying stage.	[80]
*Lactiplantibacillus paraplantarum* BPF2 and *P. acidilactici* ST6.	Starter cultures for the production of salchichones	The two autochthonous strains reduced *L. monocytogenes* counts in the samples.	[81]
*P. acidilactici* 13.	Evaluate the potential antilisterial effect when used as a starter culture.	The strain inhibited *L. monocytogenes* during sucuk fermentation.	[82]
*Ltb. curvatus* 54M16.	LAB isolated from fermented sausages for novel antimicrobial substances, producing bacteriocin(s) that are active against *L. monocytogenes*.	*Ltb. curvatus* 54M16 reduced microbial counts associated with spoilage and pathogens in traditional fermented sausages prepared without antimicrobial additives.	[83]
*Ltb. curvatus* MBSa2 and *MBSa3*	Isolation of LAB with anti-*Listeria* activity from Italian salami produced in Brazil	Strains isolated from Italian-type salami produced two bacteriocins, sakacin P and sakacin X, which were stable under heat, pH, and NaCl conditions, and inhibited *L. monocytogenes*.	[84]
*P. acidilactici* strain HA-6111-2	The combined effect of mild high-pressure processing (300 MPa) with phage P100 and *P. acidilactici* HA6111-2 as a novel decontamination method to inactivate *L. monocytogenes* in fermented meat sausages *was evaluated*.	In the Alheira model, the combination of mild high hydrostatic pressure, phage P100, and bacteriocinogenic *P. acidilactici* resulted in no detectable *L. monocytogenes* immediately after processing.	[85]
*Ltb. curvatus* MBSa2	The application of the free and entrapped strain in calcium alginate was tested for activity against *L. monocytogenes* AL602/08, a strain isolated from a meat product.	Entrapment of *Lb. curvatus* MBSa2 in calcium alginate did not affect bacteriocin production in salami.	[86]
*Ltb. sakei* ST22Ch, ST153Ch, and ST154Ch	Bacteriocins ST22Ch, ST153Ch, and ST154Ch produced by L. sakei strains ST22Ch, ST153Ch, and ST154Ch isolated from Salpicao were characterized to use these strains as co-starter bioprotective cultures in meat fermentation.	The strains exhibited antibacterial activity against multiple target microorganisms.	[87]
*Cob. alimentarius* FM-MM_4_	Purification and characterization of a novel bacteriocin produced by the bioactive strain FM-MM_4_	Lactocin MM4 was resistant to heat and a wide range of pH values. It was inactivated by proteolytic enzymes but remained active after treatment with lipase and amylase.	[65]
*P. acidilactici* HA-6111-2	Assess the combined effect of pediocin bacHA-6111-2 and mild hydrostatic pressure to control *L. innocua*.	The combination of pediocin bacHA-6111-2 and mild pressure treatments reduced *L. innocua* counts in Alheiras fermented sausages.	[88]

Overall, strain dependent variability among LAB can be explained by differences in metabolic pathways, stress tolerance, and substrate utilization. Such heterogeneity directly influences their acidification rate, bacteriocin synthesis, proteolytic capacity, and ability to dominate native microbiota under reduced-sodium conditions. The incorporation of LAB into fermented meat sausages has a positive impact on the reduction in synthetic or chemical additives, and these microorganisms have played a satisfactory role in the development of antioxidants [89]. Fernandez [71] used LAB as an alternative to inhibit the growth of *Listeria* spp. in fermented meat sausage by reducing the amount of sodium nitrite. The strains *P. acidilactici* MP14 and the commercial *P. acidilactici* B-LC-20 demonstrated efficacy in reducing *Listeria* spp., reinforcing their potential as a protective culture in nitrite-free fermented meat sausage. In addition to their direct antimicrobial activity, LAB can play a crucial role in inhibiting stress-adapted *L. monocytogenes* strains that have developed resistance to acidic or osmotic pressure environments. Given that stress adaptation enhances the survival of pathogenic bacteria, LAB can create a more restrictive environment, reducing the likelihood of persistent contamination in food matrices.

Bertuci [10] used four different strains of *Lacticaseibacillus* spp. as a biopreservative in fermented sausages. The initial assessment focused on their ability to inhibit pathogenic and spoilage microorganisms, specifically *L. monocytogenes*, *Bacillus cereus*, *Staphylococcus aureus*, and *E. coli*. Subsequently, the strains were tested for parameters such as pH and additives relevant to fermented sausages, enabling their application in a food matrix. The authors emphasized the notable ability of two commercial strains, *Lbs. rhamnosus* GG and *Lbs. paracasei* BGP1, to inhibit microorganisms and improve the product’s technological qualities.

Liu [72] evaluated the impact of Weissella species isolated from Sichuan dry sausage on the physicochemical, proteolytic, volatile, microbiological, and sensory properties of nitrite-reduced fermented sausages. Both *W. cibaria* X31 and *W. confusa* L2 demonstrated promising results, reducing the amount of nitrite (95–97%) while simultaneously ensuring microbiological quality and favorable sensory acceptance. This study revealed that these strains contributed to the “reddish” coloration of the fermented product, enhancing a desired characteristic.

In another study, given the increasing demand for clean-label alternatives, Van der Veken [73] evaluated the response of a cocktail of nontoxigenic group I *C. botulinum* to the removal of nitrate and nitrite salts in fermented sausages produced under different acidification conditions and starter culture formulations, including the application of an anticlostridial *Mammaliicoccus sciuri* strain. The results indicated limited *C. botulinum* outgrowth, even in the absence of acidification, while the anticlostridial starter culture did not enhance the inhibitory effect.

The selection of LAB from fermented products is a methodological approach designed to isolate and identify strains exhibiting specific phenotypic and genotypic traits, tailored for targeted biotechnological or industrial applications. Ji [74] isolated two strains, *L.* MPKL03 and *L.* MPKL04, which were identified as *P. pentosaceus* and *Leu. mesenteroides*, demonstrating the ability to reduce nitrite levels. The strains were observed to enhance the technological profile of the product by influencing parameters such as pH, water activity, humidity, color, and nitrite content. The study, incorporating both microorganisms, highlighted the potential of nitrite reduction and food safety enhancement.

Hu [75] conducted a prior study on 37 strains of LAB, including *Ltb. curvatus*, *Ltb. sakei*, *Weissella hellenica*, and *Lpb. plantarum*, isolated from fermented sausages, assessing growth, acidification, and antimicrobial activity. In a subsequent study [90], the most promising strains were employed to evaluate the taste potential of salt-reduced fermented sausages. *W. hellenica* and *Lpb. plantarum* exhibited satisfactory results when utilized as a starter culture, contributing to the taste enhancement of salt-reduced fermented sausages. This study investigated the compensatory role of four autochthonous LAB species—*Ltb. curvatus*, *Ltb. sakei*, *W. hellenica*, and *Lpb. plantarum*—in the physicochemical properties and taste profiles of dry sausages with 40% NaCl replaced by KCl. LAB inoculation significantly reduced pH, moisture content, and water activity while increasing organic acid and certain free amino acid (FAA) concentrations. *Ltb. curvatus*- and *Ltb. sakei*-inoculated sausages exhibited higher total acid contents. The inclusion of *Ltb. sakei* led to a 36.90% increase in sweet FAA content and a 18.18% reduction in bitter FAA content. Furthermore, ETA indicated a 5.7% reduction in bitterness response in *Llb. sakei*-inoculated sausages, which also demonstrated the most similar taste profile to traditional formulations. Li [76] highlighted the potential of *Ltb. sakei* as a starter culture to improve physicochemical parameters and mitigate flavor deficiencies in NaCl-reduced dry sausages. In this study, four LAB strains—*Llb. curvatus*, *Ltb. sakei*, *W. hellenica*, and *Lpb. plantarum*—were individually inoculated into low-sodium formulations with 40% NaCl substitution by KCl. Among these, *Ltb. sakei* was the most effective in accelerating acidification and water loss, increasing the content of sweet FAAs, and reducing bitterness, thereby compensating for taste defects associated with salt reduction. These results reinforce the applicability of *Ltb. sakei* in the development of healthier fermented sausages without compromising sensory and technological quality.

The *Lpb. plantarum* LPL-1 strain, investigated in the study by Zhang [77], played a role in decreasing the number of spoilage bacteria from the genera *Staphylococcus*, *Micrococcus*, *Enterobacteriaceae*, and *Pseudomonas* and species such as *L. monocytogenes* throughout the salami fermentation and ripening stages. This effect was evident through the reduction in pH values and the levels of tyramine, putrescine, cadaverine, and total biogenic amines in sausages. Consequently, LPL-1 emerges as a strain suitable for application in salt-reduced fermented sausages and can serve as a beneficial starter culture for the fermentation process.

Almeida [78] used models simulating the fermentation of low-sodium meat products using *Lpb.* plantarum CRL681, *Ltb.* curvatus CRL705, *Ltb.* sakei CRL1862, and *E. mundtii* CRL35. *S. vitulinus* GV318 was present in all formulations used, acting as a co-culture alongside the lactic acid bacteria strains. Positive outcomes were observed using the proteolytic strains *Ltb. curvatus* CRL705 and *S. vitulinus* GV318 under low-chloride conditions, indicating a favorable response in terms of amino acids and flavor-related peptides. Additionally, the combination of strains with low sodium concentrations may impact the cardiovascular system, owing to the limited production of peptides and free amino acids during the fermentation process.

De Oliveira [79] reported that the incorporation of BB-12 microparticles from *B. animalis* subsp. *lactis* did not affect the technological characteristics of salt-reduced salami. Lipids, color, texture profile, fatty acids, and organic acids remained unaffected. The study included three treatments: control (C), without lactic acid bacteria; B1, containing encapsulated *B. animalis* and 0.020% salt; and B2, containing the same strain with 0.010% salt. Notably, the sensory acceptance of the treatments incorporating the encapsulated LAB was also favorable. This illustrates the potential application of this strain, demonstrating its compatibility with technological and sensory parameters, and positioning it as an alternative for the development of functional fermented products with reduced salt content.

Stefan and Predescu [80] investigated the efficacy of bioprotective cultures containing *P. acidilactici*, a bacteriocin-producing strain known for its antimicrobial activity against *L. monocytogenes*. This study demonstrated that *L. monocytogenes* counts decreased by 3.2 log CFU/g over 30 days in the batch inoculated with bioprotective cultures, whereas the reduction in the control batch (without bioprotective cultures) was only 1.03 log CFU/g. These results highlight the potential of bioprotective cultures as a preventive measure to control the growth of *L. monocytogenes* in dry fermented salami, thereby contributing to enhanced food safety and reducing the risk of human listeriosis.

García-Lopez [81] evaluated the potential of two autochthonous LAB strains *Lpb. paraplantarum* BPF2 and *P. acidilactici* ST6 isolated from spontaneously fermented Spanish sausages as starter cultures for *salchichón* production at a pilot scale. These strains were assessed for their impact on physicochemical parameters, microbial composition, biogenic amine content, and sensory properties compared to a commercial starter (RAP) and a spontaneously fermented control. The results indicated that ST6 exhibited a lower final percentage in the product, whereas both BPF2 and ST6 reduced biogenic amine levels and rancidity. A challenge test confirmed their bacteriocinogenic activity against *L. monocytogenes*, demonstrating greater inhibitory efficacy than RAP and control samples.

Cosansu [82] investigated the efficacy of *P. acidilactici* 13, an autochthonous strain isolated from naturally fermented sucuk, in controlling *L. monocytogenes* during the ripening and storage of sliced turkey breast during the ripening of this dry fermented sausage. When used as a starter culture in sucuk production, *P. acidilactici* 13 reduced *L. monocytogenes* counts by 3.32 log CFU/g over an 8-day ripening period, compared with a 1.37 log CFU/g reduction in control samples. Additionally, treatment of turkey breast slices with a partially purified antimicrobial substance from *P. acidilactici* 13 led to an immediate *L. monocytogenes* reduction of 1.03 log CFU/cm^2^. These results that *P. acidilactici* 13 is a promising protective culture for controlling *L. monocytogenes* in fermented sausages.

*Ltb. curvatus* 54M16 isolated from traditional fermented sausages in Italy, exhibited the ability to produce multiple bacteriocins (sakacin X, T, and P) with antimicrobial activity against *L. monocytogenes*, *Bacillus cereus*, and *Brochothrix thermosphacta*. The strain demonstrated stability at pH 4.5 and 4% NaCl, along with strong acidification capacity, nitrate reduction, and high superoxide dismutase activity. When applied as a starter culture, it enhanced the quality and safety of fermented sausages without antimicrobial additives, highlighting its potential for clean-label meat products, as evidenced by the study of Casaburi [83].

Souza Barbosa [84] investigated the behavior of *Ltb. curvatus* MBSa2, a bacteriocinogenic strain isolated from salami, when encapsulated in calcium alginate. The performance of the strain was assessed in MRS broth and in salami artificially contaminated with *L. monocytogenes* AL602/08 over a 30-day period, simulating fermentation and maturation conditions. The entrapment process did not impair bacteriocin production, as both free and encapsulated *Ltb. curvatus* MBSa2 reduced *L. monocytogenes* counts during salami processing.

In a study by Todorov [87], *Ltb. sakei* ST22Ch, ST153Ch, and ST154Ch, isolated from traditional pork products in Northwest Portugal, demonstrated broad-spectrum antimicrobial activity against *Enterococcus* spp., *Listeria* spp., *E. coli*, *Klebsiella* spp., *Pseudomonas* spp., *Staphylococcus* spp., and *Streptococcus* spp. Bacteriocins produced were bactericidal, non-glycosylated, and heat-stable, and activity was maintained after 2 h at 100 °C. Maximum activity was observed in the early stationary phase but declined over time. Beyond their antimicrobial function, LAB also contribute to the technological and sensory development of fermented sausages through proteolysis, lipolysis, and the generation of volatile compounds.

### 3.3. Bacteriocins in Fermented Sausage Meat

The technological properties of LAB are closely related to their metabolic versatility, which influences texture, flavor, and color development in fermented sausages. The application of bacteriocins in fermented sausages provides several advantages, particularly in terms of food safety. Bacteriocins produced by LAB effectively inhibit pathogens such as *L. monocytogenes*. Notably, LAB-derived bacteriocins can also exhibit inhibitory effects against some Gram-negative pathogens and microorganisms that spoilage. However, this activity is often observed when bacteriocin is combined with surfactants, which contribute to cell wall destabilization and enhance bacteriocin effectiveness [91]. Furthermore, they serve as a natural alternative to chemical preservatives, addressing the growing consumer demand for healthier foods free from synthetic additives. Another important advantage of bacteriocins is that they do not alter the sensory characteristics of the products, such as taste, texture, or aroma, thereby preserving the sensory qualities of fermented foods [92].

Among all the studied bacteriocins, nisin, produced by *Lac. Lactis exhibits* strong antimicrobial activity against a broad spectrum of Gram-positive bacteria. Commercially, nisin is available under various trade names, including Nisaplin. Pediocin PA-1, a bacteriocin synthesized by *P. acidilactici*, demonstrates high efficacy against *Listeria* species and is commonly applied in meat. It is marketed under commercial names such as ALTA™ 2341 [93]. Bacteriocins like nisin have been shown to be highly effective in reducing pathogenic and spoilage microorganisms when applied in the food industry. However, bacteriocins are selected for specific purposes due to their limited effectiveness in the application. These processes by additives, chemical agents, and pH. These factors can alter physiological conditions and the integrity of the cell membrane, thereby affecting the interaction of bacteriocins with the target [94,95].

However, the application of bacteriocins also presents certain challenges. The spectrum of action of some bacteriocins can be limited; that is, they are effective against a restricted number of microorganisms, which may necessitate the use of supplementary preservation techniques. In addition, prolonged use of bacteriocins may promote the development of bacterial resistance, diminishing their effectiveness over time [96].

Another challenge relates to the instability of some bacteriocins under extreme processing conditions, such as high temperatures or pH variations, which can compromise their efficacy during certain production stages. The large-scale production of these substances can also be costly, potentially leading to increased food product prices. Finally, the use of bacteriocins encounters regulatory challenges because not all countries permit their use or require rigorous safety testing before approval, thereby limiting their application in specific markets [97].

Komora [85] evaluated a non-thermal multi-hurdle approach combining mild high hydrostatic pressure (HHP, 300 MPa), the bacteriophage Listex™ P100, and the pediocin PA-1-producing *P. acidilactici* HA 6111-2 to control *L. monocytogenes* in *Alheira*, a traditional fermented meat sausage from Northern Portugal. The combined treatment achieved the USDA-FSIS 5 log reduction, eliminating *L. monocytogenes* immediately after processing, whereas the dual combinations of HHP with Listex™ P100 or HHP with *P. acidilactici* were less effective. These findings highlight the potential of integrating bacteriophages, bacteriocinogenic LAB, and mild HHP as a novel strategy to enhance microbial safety in fermented sausages.

Barbosa [86] investigated the isolation of LAB with anti-*Listeria* activity from salami and characterized and semi-purified their bacteriocins to assess their effectiveness in controlling *L. monocytogenes* during salami production on a pilot scale. Two *Ltb. curvatus* strains were isolated from and identified by 16S rRNA sequencing. The bacteriocins MBSa2 and MBSa3 exhibited strong antimicrobial activity against *L. monocytogenes* and other Gram-positive bacteria. Purification analysis revealed that both strains produced two active peptides (4457.9 Da and 4360.1 Da) homologous to sakacin P and X. When the semi-purified bacteriocins from *Ltb. curvatus* MBSa2 were added to the experimentally contaminated salami batter (10^4^–10^5^ CFU/g *L. monocytogenes*), the pathogen counts were reduced by 2 log after 10 days and by 1.5 log after 20 days, demonstrating their potential to enhance salami safety during production.

The combined application of HHP (300 MPa, 5 min, 10 °C) and bacteriocin bacHA-6111-2, produced in situ or ex situ, was evaluated for its effectiveness in controlling *L. innocua* in *Alheira*, a traditional Portuguese fermented meat product. The impact of these treatments was assessed immediately after application and throughout 60 days of refrigerated storage (4 °C). Castro [88] demonstrated that a bacteriostatic effect was observed during early storage for higher initial *L. innocua* concentrations when pressure was applied alone or combined with ex situ bacteriocin production. Besides their technological relevance, certain LAB strains exhibit probiotic properties, thereby bridging the gap between food safety, product quality, and potential health benefits.

### 3.4. Probiotic LAB in Fermented Sausages

The growing interest in probiotic applications of LAB in fermented meats reflects a shift from purely technological functions toward health-oriented product innovation. There is a scientific consensus that LAB can provide benefits in biopreservative processes, and some strains can further contribute as probiotics (or postbiotics) in health-promoting benefits. This approach is rooted in microbial antagonism, employing nonpathogenic strains as protective cultures in food due to their ability to generate antimicrobial substances [98]. Moreover, when probiotic LAB is used in fermented sausages, it can modulate the microbiota of the consumers, thereby promoting additional health properties. The benefits of this treatment include improvement of gastrointestinal balance, stimulation of the immune system, and competition with some pathogens [99].

In general, the health benefits of probiotic LAB are based on the strains’ selection and viability, their adaptation to the food environment, and their resistance to the gastrointestinal tract, thereby generating health benefits for the consumer [100]. Fermented meat sausages do not require heat treatment, making them ideal for probiotic strain application. These meat products are hypothesized to effectively protect LAB under gastrointestinal conditions [101]. However, there is a potential negative impact on the cell viability of the probiotic strains when applied to meat products due to reactions, such as fermentation and drying, that occur in the product [102].

In contrast, fermented meat sausage contains high amounts of NaCl (2–4% total product weight), nitrite, and nitrate, which reduce the pH to 4.5 and water activity to 0.90 [103]. Therefore, the potential probiotic strains must be able to thrive in this harsh environment. The selection of strains with these capabilities is fundamental for their application in fermented sausages, due to the characteristics required for the fermented meat matrix process [104]. Some strains of BAL, such as *Bifidobacterium longum* KACC91563; *E. faecium* CECT410; *Lbs. casei* SJRP66; *Lbs. paracasei* DTA83; *Lbs. rhamnosus LOCK900*; *Lpb. plantarum* 299v; *Lactobacillus acidophilus* CRL1014; *Ltb.* sakei 23K; *Limosilactobacillus fermentum* R6; and *Staphylococcus simulans* NJ201, have potential applications in fermented sausages. The production of fermented meat sausage with probiotics shows their successful colonization of the meat matrix and their ability to maintain high counts throughout processing. This is attributed to their resistance to NaCl and nitrate/nitrite salts, with minimal effects on product quality, such as color, pH, oxidative status, texture, and sensory attributes. However, human studies using probiotic LAB strains in fermented sausages are still scarce [96,97].

When selecting LAB strains with probiotic potential for application in meat products, various factors must be assessed through both in vitro and in vivo analyses to ensure that the selected strains can effectively survive and function within both the meat matrix and the human digestive system [105]. Survival in the GIT is critical, with a focus on resistance to pH fluctuations, digestive enzymes, salts, and interaction with other members of the host microbiome. Furthermore, the strains must be able to adapt to the distinctive characteristics of the meat environment, which include high protein content, specific water activity (aw) levels, moisture content, and fats. LAB are particularly well-suited for meat fermentation because of their capacity to facilitate acidification, flavor and texture development, and color stabilization. Additionally, the probiotic efficacy of LAB in meat products must be demonstrated through their antimicrobial activity and low resistance to antibiotics, ensuring both product safety and health benefits for consumers [106].

Liu et al. [107] evaluated the antimicrobial and antioxidant activities of *Ltb. sakei*, *Lpb. plantarum*, and *P. pentosaceus* and their applicability as starter cultures in sausage fermentation. When applied individually or in combination, these strains exhibited significant inhibitory effects against *E. coli* and *S. aureus*, along with pronounced antioxidant capacity, as evidenced by DPPH, ABTS, and hydroxyl radical scavenging activities, reducing power, and elevated antioxidant enzyme activities. Moreover, the inoculation of probiotic LAB maintained the physicochemical and sensory attributes of naturally fermented sausages while improving color and texture. Among the tested strains, *Lpb. plantarum* notably yielded higher sensory values.

## 4. Conclusions

The reformulation of fermented sausages through the reduction of sodium chloride and other curing agents remains a significant technological and safety challenge, particularly in the context of increasing consumer demand for healthier, clean-label products. Maintaining product integrity, microbiological safety, and sensory quality is essential, and LAB represents a multifunctional approach to address these objectives. LAB can function as bioprotective cultures, inhibiting spoilage and pathogenic microorganisms, while certain strains also confer probiotic properties, providing additional health benefits. Future research should prioritize the systematic selection and characterization of LAB strains capable of delivering both technological and probiotic functions under reduced-sodium conditions. Evaluating strain performance across diverse meat matrices, investigating interactions with natural preservatives and flavor modulators, assessing long-term effects on sensory attributes and consumer acceptance, and optimizing starter culture combinations and inoculation levels to balance microbial safety, product quality, and functional properties. Advancing these aspects will generate actionable knowledge to guide the development of safer, nutritionally optimized, and consumer acceptable fermented sausages.

## Data Availability

No new data were created or analyzed in this study. Data sharing is not applicable to this article.
